# Facilitators and Strategies for Breaking the News of an Intrauterine Death—A Mixed Methods Study among Obstetricians

**DOI:** 10.3390/jcm10225347

**Published:** 2021-11-17

**Authors:** Dana Anais Muin, Janina Sophie Erlacher, Stephanie Leutgeb, Anna Felnhofer

**Affiliations:** 1 Department of Obstetrics and Gynecology, Division of Fetomaternal Medicine, Comprehensive Center for Pediatrics Medical University of Vienna, Waehringer Guertel 18-20, 1090 Vienna, Austria; n01621749@students.meduniwien.ac.at; 2 Austrian Society of Obstetrics and Gynecology, Frankgasse 8, 1090 Vienna, Austria; stephanie.leutgeb@oeggg.at; 3 Department of Pediatrics and Adolescent Medicine, Division of Pediatric Pulmonology, Allergology and Endocrinology, Comprehensive Center for Pediatrics Medical University of Vienna, Waehringer Guertel 18-20, 1090 Vienna, Austria; anna.felnhofer@meduniwien.ac.at

**Keywords:** stillbirth, fetal death, intrauterine death, breaking bad news, resilience, stress, coping strategies

## Abstract

(1) Background: The death of a baby in utero is a very sad event for both the affected parents and the caring doctors. By this study, we aimed to assess the tools, which may help obstetricians to overcome this challenge in their profession. (2) Methods: We conducted a cross-sectional online survey in 1526 obstetricians registered with the Austrian Society of Obstetrics and Gynecology between September and October 2020. (3) Results: With a response rate of 24.2% (*n* = 439), our study shows that diagnosing fetal death was associated with a moderate to high degree of stress, regardless of position (*p* = 0.949), age (*p* = 0.110), gender (*p* = 0.155), and experience (*p* = 0.150) of physicians. Coping strategies for delivering the news of intrauterine death to affected parents were relying on clinical knowledge and high levels of self-confidence (55.0%; 203/369), support from colleagues (53.9%; 199/369), and debriefing (52.8%; 195/369). In general, facilitators for breaking bad news were more commonly cultivated by female obstetricians [OR 1.267 (95% CI 1.149–1.396); *p* < 0.001], residents [χ^2^(3;369) = 9.937; *p* = 0.019], and obstetricians of younger age [41 (34–50) years vs. 45 (36–55) years; *p* = 0.018]. External facilitators were most frequently mentioned, including professional support, training, professional guidance, time, parents’ leaflets, follow-up consultations, a supporting consultation atmosphere, and preparation before delivering the bad news. Internal facilitators included knowledge, empathy, seeking silence, reflection, privacy, and relief of guilt. (4) Conclusions: Communicating the diagnosis of fetal death evokes moderate to high levels of stress among obstetricians. Resources from both the professional and private environment are required to deal with this professional challenge on a personal level.

## 1. Introduction

In high-income countries, the rate of antepartum stillbirth, or intrauterine fetal death (IUFD) above 22 weeks of gestation, ranges between 2.6 to 9.1 per 1000 births [1]. Despite its relatively small and stable prevalence in high-income countries, fetal death has an immeasurable and profound impact on the personal and intimate life of affected parents. In addition, from the perspective of the caring physician, detecting and/or communicating the diagnosis of intrauterine death has a personal impact with regard to stress and burden [2]. In semi-structured in-depth qualitative interviews with eight consultants, Nuzum et al. found that two super-ordinate themes dominate obstetricians’ state following the diagnosis of stillbirth: the weight of experienced professional responsibility, and their personal human response to stillbirth [3]. Clearly, stillbirth was identified as one of the most difficult parts of their jobs, whilst, paradoxically, none of the physicians had received any specialist training in perinatal bereavement care, as all learned “on the job” and from senior colleagues during their training years. Physicians also noted that recalling these particular situations during the interview opened up “painful and vivid memories”. Additionally, the burden of stillbirth experience was found to be unwaveringly high over time, with lasting resentments expressed as “sadness, fear, anger, disappointment and personal grief” [3,4].

Causes of intrauterine death are diverse, and in the majority of cases, unclear at the time of diagnosis [5]. Circumstances that lead women to seek the doctor’s or midwife’s office may be acute, such as bleeding or pain, or within the frame of a routine check-up. Often, however, women feel that “something is wrong”, either by intuition, or by reduced or increased fetal movements [6,7]. Diagnosis of fetal death is usually made by real-time ultrasonography and confirmed upon absent fetal heart beats and blood circulation in the umbilical cord. For the attending physician, breaking news of fetal death requires a sensitive approach and an empathetic communication towards the affected parents [8]. The physician’s confrontation with fetal death and care for the bereaved may be over-whelming, especially in case of perceived lack of training and limited support.

By means of this mixed methods study among Austrian obstetricians, we, therefore, sought to assess the level of stress which obstetricians experience when diagnosing fetal death and delivering the news to the parents. We, furthermore, aimed to explore factors that may facilitate this situation of breaking such bad news. Lastly, we set out to identify physicians’ individual strategies that support them in coping with these and other professional challenges in obstetrics.

## 2. Materials and Methods

### 2.1. Data Collection

An online survey was conducted via SurveyMonkey (https://www.surveymonkey.de/ (accessed on 4 November 2020)), a standard tool for online surveys, which allows for collecting data anonymously without storing sensitive background information (i.e., IP-address). The target population was 1526 Austrian obstetricians and gynecologists registered with the Austrian Society of Obstetrics and Gynecology (Oesterreichische Gesellschaft fuer Gynaekologie und Geburtshilfe; OEGGG). The survey link was sent out with an invitation email via the OEGGG email-server between 21 September 2020 and 31 October 2020. Authorized access to the survey data source and email-list was solely and confidentially provided to the OEGGG secretary (S.L.). Two friendly reminders were sent out by weeks 2 and 3. No incentives were offered, and participants were informed that the data would be published and presented at the annual meeting of the society.

The survey questionnaire was conceived by the study team (D.A.M., J.S.E., A.F.) and approved by the Medical Board of the OEGGG as well as the Ethics Committee of the Medical University of Vienna. Usability and technical functionality of the electronic questionnaire were tested among the study team and with five voluntary participants before fielding the questionnaire. Reliability was established by an oral interview of the five participants after the completion of the survey, in which the survey questions were repeated. The variance between written and oral responses was 0.1%.

Participants were informed about the purpose of the study, the investigators, the anonymity of their data and the approximate length of the survey (15 min). The survey adhered to the Declaration of Helsinki, and an online informed consent by a tick-box was obtained upon digital participation.

The total online questionnaire entailed seven pages with four to five questions per page. Respondents were able to review and change their answers via a return button. Consistency or completeness checks were not included before signing-off. The closed survey was protected against un-authorized access. All received responses were anonymous, and no direct personal information were collected or stored. No cookies were used in this survey. Duplicate entries were avoided by preventing users’ access to the survey twice. Time to fill in a questionnaire was not assessed by the investigators. No methods were used to adjust for potential non-representative samples. Only completed questionnaires were analyzed. Collected data were transferred into an Excel file sheet and checked for integrity and consistency.

### 2.2. Measures

For this survey, we constructed a mixed methods research design, integrating quantitative and qualitative data derived from an online 16-item questionnaire, which included three validated questionnaires, overarching in total eight domains: (a) demographic data, (b) experience, (c) stress coping [9,10], (d) coping strategies, (e) open-answer textbox regarding facilitators, (f) trait empathy [11,12], (g) locus of control [12,13] and (h) affect. For the purpose of this study, we extracted data from domains (a–e) only. Data and results of the other domains are presented elsewhere (Muin et al., manuscript under revision).

Demographics

The following key demographic variables were assessed as categorical variables: gender, age, children, marital status, current position, year of residence, and current workplace (e.g., university hospital vs. private practice).

Experience

Participating obstetricians were asked to categorize their level of experience in having previously diagnosed and delivered the news of fetal deaths to affected parents (i.e., “0”, “<5”, “6–10”, “11–30” and “>31” times).

Stress Perception

Participants were asked to rate the question “The situation was stressful for me” (i.e., perceived stress) when delivering the diagnosis of fetal death, on a 5-point-Likert-scale (1 = does not apply, 5 = fully applies) [9,10].

Coping Strategies

Obstetricians were asked to grade which strategies (i.e., activities or attitudes) were most useful to them for coping (a) with the circumstance of diagnosing or breaking news of fetal death, and (b) with challenging obstetrical situations in general.

The selection of variables was based upon the conceptual model of resilience in health-care professionals, encompassing the following values and items: social culture, personal life, individual identity, professional identity, professional community, and medical culture [14].

The participants could choose between the categories (a) “Clinical knowledge and self-confidence”, “Team based decisions”, “Psychological support”, “Psychological debriefing after adverse events”, “Balint groups”, “Supervision”, “Support from your supervisor/head of department”, “Support from colleagues”, “No strategy”, and “Others”, and (b) “Conversation with colleagues”, “Conversation with my partner”, “Follow-up consultations with parents”, “Balint groups”, “Distraction or avoidance”, “Psychotherapy”, “Antidepressant medication”, “No strategy”, and “Others”, respectively.

Open Responses

Finally, a single text box for open responses was placed at the end of the questionnaire to the question “What do you think may help to facilitate this situation for you?” The answers from this open-ended question were collected, harmonized into main themes and divided into two categories by inductive approach for further analyses [15,16]. Individual items were counterchecked for plausibility and accuracy by all co-authors.

Facilitators were defined as factors, which help an individual to overcome a situation, rebuild and regain their strength for further professional performance and mitigate stress. Internal facilitators were defined as endogenic factors, i.e., values, attitudes or habits derived from within and cultivated by an individual person. External facilitators were defined as outer circumstances that can be either provided or acquired by the environment or by one-self in order to support an experience, such as the workflow, procedures or professional habits.

### 2.3. Definitions

Residents in obstetrics and gynecology are part-time or full-time working hospital doctors in specialist training for the duration of six to eight years in Austria. Routine duties include seeing patients in outpatient clinic, participating in ward rounds, theatre and delivery units, as well as doing on-calls and nights shifts.

Specialty doctors in obstetrics and gynecology working full- or part-time in hospital are considered equivalent to consultants. Their duties range from leading ward rounds, deliveries, theatres and outpatient clinics according to their special fields of interest, as well as doing on-calls and night shifts. They may have an additional part-time private practice outside the hospital; however, they were considered as “specialist doctors in hospitals” only.

Departmental heads are leading specialty doctors who are in charge of a department of obstetrics and/or gynecology and/or reproductive medicine, who usually work full-time in hospital and are on standby for emergencies during nights. They often lead outpatient clinics and may see their own patients in an affiliated private clinic or in hospital.

Specialty doctors working only in private or public funded practice are hospital-independent obstetricians and gynecologists with flexible working hours and an individual emphasis on obstetrics and/or gynecology. They are the first to be consulted by women with a gynecological problem or emergencies outside hospital. They also provide annual gynecological check-ups, carry out regular routine scans in pregnant women, and fetal and maternal well-being examinations.

### 2.4. Statistical Analyses

Distribution of data was analyzed using the Kolmogorov–Smirnov test. Categorical data are given as absolute (n) and relative frequencies (%). Continuous data are given as mean (M) and standard deviation (SD) or median and 25th and 75th percentile. Categorical data were compared with Chi^2^ and Fisher’s Exact test, respectively. Continuous data were compared with a Kruskal–Wallis test with Dunn’s multiple comparison test. Univariate Analyses of Variance (ANOVAs) were used to analyze group-differences with ordinal variables. All reported *p*-values are two-sided, and a *p*-value < 0.05 was considered as statistically significant. Statistical tests were performed with SPSS Statistics Version 26 (IBM Corporation, Armonk, NY, USA). Figures were designed by GraphPad Prism 9 for macOS (GraphPad Software, LLC, San Diego, CA, USA). The Venn diagram was manually designed using Graphic for Mac to illustrate a theme cloud: The circle sizes correlate with the frequency of a mentioned element in the open-ended questions, and the circle colors represent the predominant professional group to mention this element.

## 3. Results

### 3.1. Baseline Characteristics

**Figure 1:**
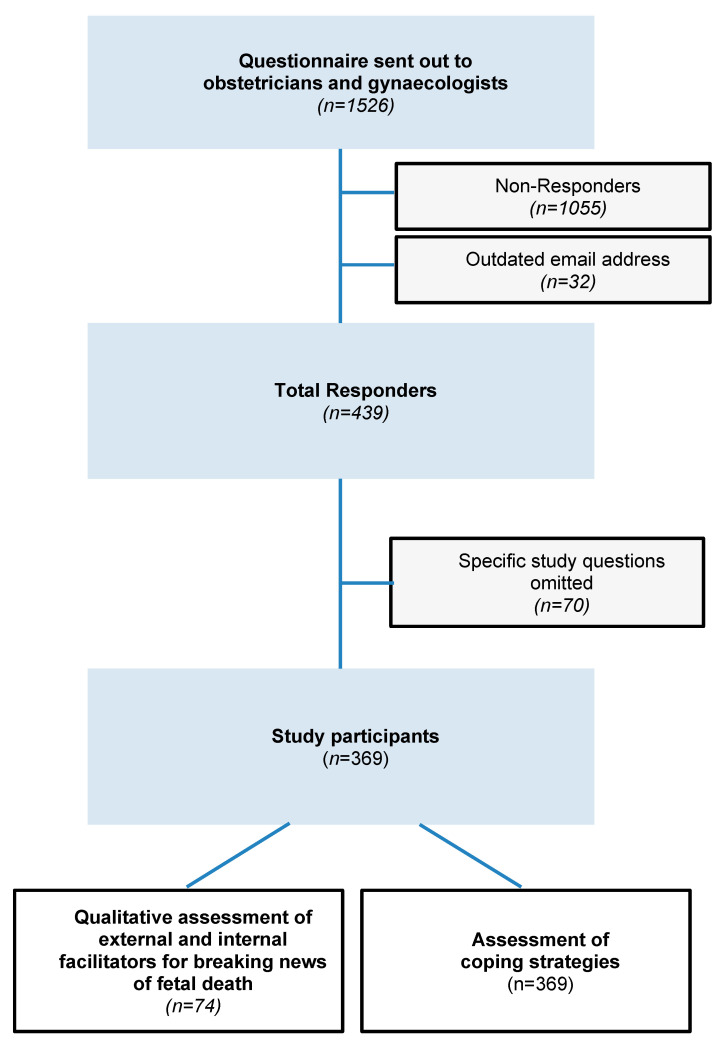
Flowchart illustrating the enrolment of participants (*n* = 369) to the survey conducted by the Austrian Society of Obstetrics and Gynecology between 21 September 2020 and 31 October 2020.

In total, 439 obstetricians and gynecologists completed the online survey (Figure 1).

For this study and due to missing responses, we included 369 participants (*n* = 88 residents; *n* = 129 hospital specialist doctors; *n* = 21 departmental heads; *n* = 131 specialist doctors working in private or public practice), all of who answered the question regarding coping strategies. Seventy-four were included into the qualitative assessment of facilitators for breaking news of fetal death. Median age of participants was 44 (36–54) years with a female participants’ rate of 76.4%. The baseline characteristics of all participants are shown in Table 1.

### 3.2. Main Parameters

#### 3.2.1. Experience

Most participants indicated to have diagnosed and delivered the news of fetal death up to five times (*n* = 164/369; 44.4%, and *n* = 151/369; 40.9%, respectively), whereas departmental heads showed to have acquired significantly more experience compared to other professional groups [F(3, 365) = 3.893, *p* = 0.009; and F(3, 365) = 3.769, *p* = 0.011, respectively; Table 1)].

#### 3.2.2. Stress

**Figure 2:**
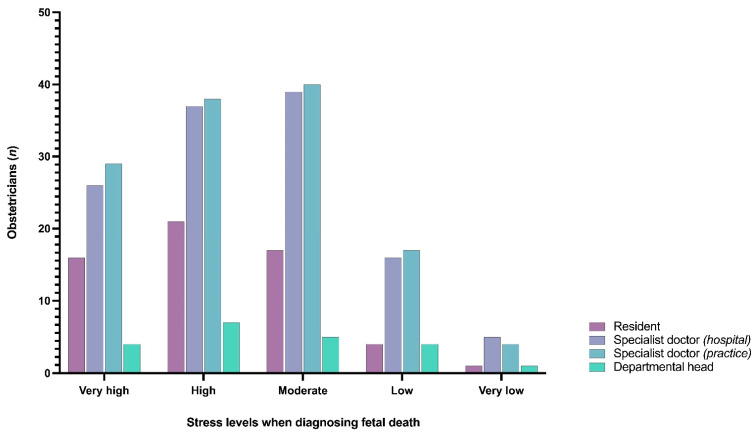
Clustered bar chart on stress levels (i.e., very high; high; moderate; low; very low) in obstetricians when diagnosing fetal death.

Figure 2 illustrates the distribution of self-reported stress levels among obstetricians per position and shows that diagnosing fetal death is associated with moderate to high degrees of stress among all participants, regardless of position [M = 3.08, SD = 0.903; F(4, 326) = 0.180, *p* = 0.949], age [M = 45.91, SD = 10.88; F(4, 311) = 1.901, *p* = 0.110], gender [M = 1.26, SD = 0.438; F(4, 326) = 1.676, *p* = 0.155], and experience [M = 3.55, SD = 1.697; F(4, 326) = 1.698, *p* = 0.150].

#### 3.2.3. Coping Strategies for Breaking News of Fetal Death

**Figure 3:**
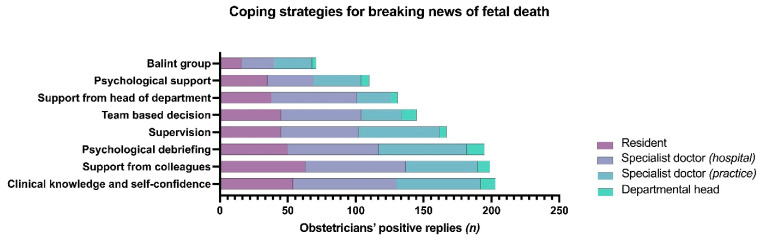
Clustered bar chart on the frequency of coping strategies among obstetricians for breaking news of fetal death.

The majority of obstetricians consider clinical knowledge and self-confidence the most useful strategy for coping with delivering the news of fetal death (*n* = 203/369; 55.0%). The second and third most prevalent coping tools were support from colleagues (*n* = 199/369; 53.9%), and debriefing (*n* = 195/369; 52.8%; Figure 3).

#### 3.2.4. Coping Strategies in Stressful Obstetrical Events

**Figure 4:**
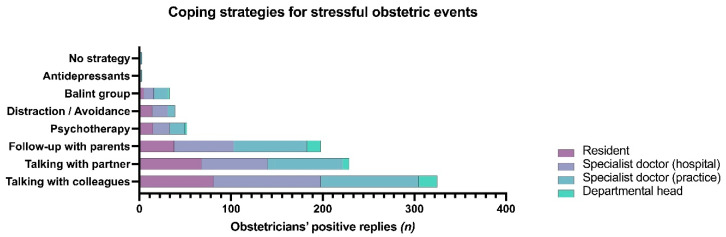
Clustered bar chart on the frequency of strategies among obstetricians for coping with stressful obstetric events.

As to coping with professional challenges in obstetrics in general, the majority of obstetricians consider talking to colleagues as the most useful strategy (*n* = 325/369; 88.1%), followed by talking to one’s spouse or partner (*n* = 229/369; 62.1%), and a follow-up consultation with parents (*n* = 198/369; 53.7%). A minority of obstetricians indicated to consume antidepressants or have no strategy at all (each *n* = 3/369; 0.8%; Figure 4).

#### 3.2.5. Facilitators for Delivering News of Fetal Death

**Figure 5:**
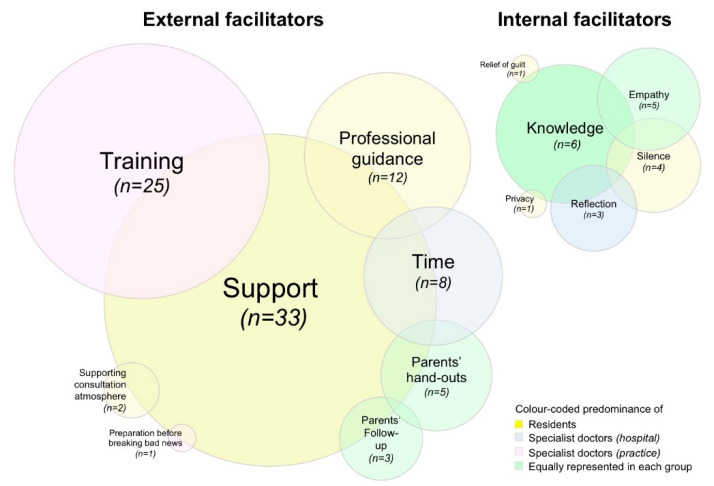
Graphical theme cloud illustrating obstetricians’ external and internal facilitators for breaking news of fetal death to affected parents. The size of the circles directly correlates with the number of positive replies by obstetricians. The color of the circles represents the respective professional group, which predominantly quoted this element in the open response (i.e., residents; specialist doctors in hospital and practice, respectively; departmental heads).

Facilitators were more commonly present in women [OR 1.267 (95% CI 1.149–1.396); *p* < 0.001] and residents [χ^2^(3) = 9.937; *p* = 0.019] and therefore obstetricians of younger median age [41 (34–50) years vs. 45 (36–55) years; *p* = 0.018]. From 74 responses, we distilled professional and personal values into a theme cloud of both external and internal facilitators to help break the news of fetal death for obstetricians (Figure 5).

In total, external facilitators most frequently resonated with participants, especially among residents. These entailed the values in professional culture, i.e., professional support, training, professional guidance, time, parents’ leaflets, follow-up consultations, a supporting consultation atmosphere, and lastly, preparation before delivering the bad news. Sample narratives are presented in Table 2 to illustrate each theme.

Internal facilitators, that support breaking bad news of fetal death, entailed the motives surrounding personal culture, i.e., knowledge/wisdom, empathy, silence, reflection, privacy and relief of guilt. Sample narratives are presented in Table 3 to illustrate each theme.

## 4. Discussion

### 4.1. Main Findings

In this cross-sectional online survey, we found that diagnosing fetal death evokes moderate to high levels of stress in obstetricians, regardless of experience, position, age, or gender. When trying to cope with stress of breaking news of fetal death, obstetricians orient their needs towards different sources depending on their level of experience and position: Whilst residents more commonly turn to colleagues for help and support, departmental heads cope with stress by team debriefing. Specialist doctors in hospital and private or public practice, however, ground their stability through acquisition of expertise and clinical knowledge. Likewise, with regards to facilitators, the needs are individually directed as per role and position: In general, more externally-mediated facilitators were identified, with the most prevalent ones being “support” and “professional guidance”, which, yet again, were most commonly requested by residents.

### 4.2. Results in the Context of What Is Known

Experiencing stillbirth as a bereaved parent is a traumatic event that requires careful and empathetic communication [17,18]. Previous studies have shown that stillbirth has an unacknowledged impact on obstetricians as well, which has been neglected in education and psychological support at the workplace [3]. Our findings support previous qualitative survey studies highlighting the need for additional training and the value of peer support from colleagues [3,19,20]. In 2008, Gold et al. explored experiences and attitudes about perinatal death, as well as coping strategies and training by an online survey among 804 obstetricians in the United States (U.S.) [20]. The authors found that the majority of respondents agreed that detecting stillbirth “took a large emotional toll” on them personally. Adequate training to cope with fetal death was noted to significantly mitigate the feelings of guilt, worries about legal actions, and the consideration of giving up obstetrics all together. In addition, most common coping strategies were found to be “talking to colleagues” (87%) or one’s “friends” (56%).

These results are also in accordance with the findings of Farrow et al., who conducted a U.S. questionnaire survey on the psychological impact of stillbirth and the influence of epidemiological factors in stillbirth reactions among 499 obstetricians in 2013 [19]. The authors specifically explored the spectrum of physicians’ psychological responses towards a pregnancy, which ended in stillbirth, and found that, overall, grief was the most common emotional response to stillbirth (53.7%), followed by self-doubt (17.2%). Of note, the authors found that older physicians (≥51 years of age) and physicians in solo and private practice were more likely to suffer from depression, as they might feel more isolated and experience greater lack of support from colleagues to process stillbirth [19].

### 4.3. Clinical Implications

To our knowledge, our study is the first to specifically explore obstetricians’ attitude and coping strategies when confirming and delivering the burdensome diagnosis of fetal death. More so, our data together with the findings from the previous surveys conducted by Gold and Farrow et al. [19,20] suggest that the psychological impact of fetal death on physicians is not to be ignored and that a sustainable social and peer network are of importance for coping with professional challenges.

“Obstetrics” is a term that emerged in the mid 18th century, derived from the modern Latin word *obstetrix*, meaning ‘*midwife*’ and *obstare* ‘to be *present*’. Indeed, obstetrics, as taught today at medical schools in the western world, primarily focuses on the science and knowledge regarding maternal and fetal care before, during and after childbirth. The prime intention of reproduction and pregnancy is to help to give birth to a healthy infant, with all its biological, social, cultural roles and expectations attached to and surrounded by [21]. The anticipation of a vital new-born—as grounded in our human nature by parental archetypes—marks a strong drive within the medical profession of obstetrics and midwifery, so that the contrary of such—the delivery of a dead infant—seems to erratically run against human instincts and causes disturbance and rejection on deep layers of medical professionalism and psychological identification of the individuals involved in its care. This phenomenon is reflected not only by the lack of relevant chapters on fetal death in numerous obstetric textbooks, yet also in the lack of consideration of respective teaching elements within the medical curriculum and during specialty training on how to break news of fetal death. The results of our study reflect this lack by flagging up the degree of stress in this population and the need for better training and education in that subject, biologically, psychologically and in terms of patient- and topic-centered-communication skills.

The design and introduction of a specific learning tool for clinicians “IMproving Perinatal Mortality Review and Outcomes Via Education” (IMPROVE; https://learn.stillbirthcre.org.au (accessed on 1 October 2021) showed to increase confidence and knowledge of healthcare professionals in managing perinatal deaths [22]: It delivers a structured and integrated clinical and problem-oriented learning on all domains of stillbirth (communication, post-mortem examination, classification, audit and bereavement care).

### 4.4. Research Implications

Learning tools, such as the IMPROVE workshop [22], may also support clinicians with managing stress surrounding diagnosis and communication. Specific training programs ought to be revised and implemented at institutions, which care for bereaved parents after fetal loss, the content of which should entail the medical, social and psychological impact of stillbirth, as well as techniques for enhancing communication skills and obstetricians’ resilience encountering death in medicine. Subsequent steps to quantitatively and qualitatively assess parents’ and physicians’ experiences would close the audit loop and allow further improvement.

### 4.5. Strengths and Limitations

The paucity of data regarding the impact of diagnosing and delivering the diagnosis of fetal death on health care professionals, and the scarce body of evidence regarding resilience among obstetricians, justify our study and provide validity to our data to add to the current literature. In addition, our study is unique to have specifically examined the level of self-reported stress among attending physicians and their means of coping and facilitators at diagnosing and delivering the news of fetal death. The response rate of about 24.2% of participants is consistent with current trends for social science surveys administered through the internet [23]. The range of demographic and professional characteristics in responders reflects the diversity of physicians involved in stillbirth care.

After all, our study is not devoid of limitations inherent to the failure to control for recall bias of responders and thus data accuracy from returned questionnaires. We also acknowledge a potential response and selection bias by obstetricians with either greater interest in stillbirth or previous unfavorable experiences in diagnosing or communicating fetal death. Furthermore, our study did not assess the amount of time, which had elapsed since the responder’s last experience of stillbirth, which might have made a difference with regards to the individual perception of stress. In addition, we retrospectively assessed the levels of stress and coping strategies, relying on the participants’ memory. Hence, we cannot preclude a certain recall bias. Finally, our survey was conducted in a European high-income country. We, therefore, note that our data might not be fully generalizable due to potential fundamental differences in health care systems and governance, thus medical conduct and practice, cultural and social behavior, as well as teaching systems at medical school and during residency. After all, this limitation highlights the need for further data generation on that matter.

## 5. Conclusions

Our study shows that engagement in stillbirth evokes moderate to high levels of stress among obstetricians regardless of prior experiences, professional position, age, and gender. Handling these situations requires resources from both the professional and private environment, the context of which differs with the grade of professional experience and role.

## Figures and Tables

**Table 1:** Baseline characteristics of obstetricians who participated in the online survey conducted by the Austrian Society of Obstetrics and Gynecology between 21 September 2020 and 31 October 2020 (*n* = 369).

		Total (*n* = 369)	Resident (*n* = 88)	Specialist Doctor in Hospital (*n* = 129)	Departmental Head (*n* = 21)	Specialist Doctor in Private/Public Practice (*n* = 131)	*p*-Value
Age (*n* = 351)	(Median; min-max; in years)	44 (24–67)	31 (24–42)	43 (26–66)	57 (45–64)	52 (26–67)	
Sex (*n* = 369)	Female	282 (76.4%)	80 (90.9%)	99 (76.7%)	5 (23.8%)	98 (74.8%)	
	Male	87 (23.6%)	8 (9.1%)	30 (23.3%)	16 (76.2%)	33 (25.2%)	
Marital status (*n* = 364)	Single	52 (14.3%)	17 (19.5%)	24 (19.2%)	2 (9.5%)	9 (6.9%)	
	Coupled	71 (19.5%)	34 (39.1%)	19 (15.2%)	1 (4.8%)	17 (13%)	
	Married	228 (62.6%)	35 (40.2%)	77 (61.6%)	18 (85.7%)	98 (74.8%)	
	Divorced	13 (3.6%)	1 (1.1%)	5 (4.0%)	0 (0.0%)	7 (5.3%)	
Parent (*n* = 367)	Yes	253 (68.9%)	31 (35.2%)	97 (75.8%)	19 (90.5%)	106 (81.5%)	
	No	114 (31.1%)	57 (64.8%)	31 (24.2%)	2 (9.5%)	24 (18.5%)	
Diagnosed IUFD (*n*)	0	39 (10.6%)	27 (30.7%)	7 (5.4%)	0 (0.0%)	5 (3.8%)	χ^2^ (12; *N* = 369) = 114.821; *p* < 0.001
	<5	164 (44.4%)	51 (58%)	51 (39.5%)	3 (14.3%)	59 (45.0%)	
	6–10	76 (20.6%)	9 (10.2%)	31 (24%)	1 (4.8%)	35 (26.7%)	
	11–30	53 (14.4%)	1 (1.1%)	22 (17.1%)	9 (42.9%)	21 (16%)	
	>31	37 (10%)	0 (0%)	18 (14%)	8 (38.1%)	11 (8.4%)	
Delivered diagnoses of IUFD (*n*)	0	42 (11.4%)	34 (38.6%)	5 (3.9%)	0 (0.0%)	3 (2.3%)	χ^2^ (12; *N* = 369) = 147.043; *p* < 0.001
	<5	151 (40.9%)	45 (51.1%)	47 (36.4%)	2 (9.5%)	57 (43.5%)	
	6–10	82 (22.2%)	7 (8.0%)	36 (27.9%)	2 (9.5%)	37 (28.2%)	
	11–30	53 (14.4%)	2 (2.3%)	21 (16.3%)	8 (38.1%)	22 (16.8%)	
	>31	41 (11.1%)	0 (0.0%)	20 (15.5%)	9 (42.9%)	12 (9.2%)	

Abbreviations: IUFD, intrauterine fetal death.

**Table 2:** Sample narratives by obstetricians expressing their spectrum of external facilitators when delivering news of fetal death.

External Facilitator (Listed per Frequency)	Exemplar Quotes (Translated from German to English)
Support	*“To have the opportunity to talk to my colleagues regarding this situation”* (Female resident, 24 y/o, single, diagnosed IUFDs < 5 times)
	*“To have support from experienced colleagues and professionals dealing with crisis-intervention”* (Female 3rd year resident, 34 y/o, single, never diagnosed IUFD)
	*“To know within the team what and how to break the bad news (with all residents, specialist doctors, midwives) and also debrief in this team after the consultation”* (Female specialist doctor in private practice, 53 y/o, coupled; diagnosed IUFDs 6–10 times)
Training	*“To have continuous trainings and skills-and-drills simulation practice”* (Female specialist doctor in private practice, 45 y/o, married; diagnosed IUFDs 11–30 times)
	*“This situation will always be terrible, whatever the circumstance. However, frequent courses help and foster reflective practice”* (Female specialist doctor in public practice, 42 y/o; married, diagnosed IUFDs < 5 times)
	*“To gain knowledge on how to handle these consultations by learning from experienced colleagues, psychologists,* etc. *You will never feel good during these consultations. It would be advisable to have predefined intern standards, on what needs to be checked post-mortem”* (Male specialist doctor in public practice, 50 y/o, coupled; diagnosed IUFDs < 5 times)
Professional guidance	*“It would be helpful to have a short guidance or checklist with all points that have to be raised within such consultation and what needs to be considered”* (Female 1st year resident, married, never diagnosed IUFD)
Time	*“To have time during clinics, to be there for the patient and also for oneself to debrief after such consultation”* (Female consultant, diagnosed IUFDs > 31 times)
Parents’ handouts	*“Professional handouts and leaflets for the parents”* (Female specialist doctor in pubic practice, 43 y/o; divorced, diagnosed IUFDs 6–19 times)
Parents’ follow-up	*“It would be helpful to receive feedback from affected women to understand what went well or not so well during these consultations, and what they wished to be different next time”* (Female consultant, 55 y/o; married, diagnosed IUFDs < 5 times)
Supporting consultation atmosphere	*“To lead this consultation with another colleague, to be stronger together”* (Female 6th year resident, 35 y/o; married, diagnosed IUFDs 6–10 times)
Preparation	*“Briefing and debriefing: Being mentally and verbally prepared what to say and how to act”* (Female specialist doctor in public practice, 35 y/o; married, diagnosed IUFDs 6–10 times)

**Table 3:** Sample narratives by obstetricians expressing their spectrum of internal facilitators when delivering news of fetal death.

Internal Facilitator (Listed per Frequency)	Exemplar Quotes (Translated from German to English)
Knowledge	*“To know and understand what needs to be done after fetal death (e.g., genetic testing), to know what, when and how to take all necessary samples and tissues. To know how to advice and consult parents after the diagnosis.”* (Female 2nd year resident, 31 y/o; coupled, diagnosed IUFDs < 5 times)
Empathy	*“To be fully empathetic with the patient and be medically well trained and skilled”* (Male specialist doctor in private practice, 62 y/o; married, diagnosed IUFDs 11–30 times)
Silence	*“Time, silence and willingness to reflect for oneself after such event. Be centered and mindful.”* (Female specialist doctor in public practice, 42 y/o; married, diagnosed IUFDs < 5 times)
Reflection	*“This situation will always be terrible; regular training and reflection are helpful”* (Female consultant, 42 y/o; married, diagnosed IUFDs 6–10 times)
Privacy	*“Privacy; to have my other half by my side”* (Female 1st year resident, 26 y/o; Single, diagnosed IUFDs < 5 times)
Relief of professional guilt	*“Somebody to tell me that I am not responsible for the adverse outcome”* (Female consultant, 38 y/o; coupled, diagnosed IUFDs > 31 times)

## Data Availability

The data that support the findings of this study are available from the corresponding author, D.A.M., upon reasonable request.
